# Supplementary material for: Integrating protein language and geometric deep learning models for enhanced vaccine antigen prediction
Source: Nat Commun. 2025 Dec 21;17:1033. doi: 10.1038/s41467-025-67778-2 (PMC12847940; doi:10.1038/s41467-025-67778-2)
Supplement: Supplementary file 1 — Supplementary Information [file 41467_2025_67778_MOESM1_ESM.pdf]

Supplementary

Integrating Protein Language and Geometric Deep Learning Models for Enhanced Vaccine Antigen Prediction

Xiaodong Zai[1†], Yunxiang Zhao[1†], Xiaolin Wang[1], Mingyue Leng[2] , Menglong Lu[2], Yilong Yang[1], Xiaofan Zhao[1], Ruihua Li[1], Yaohui Li[1], Yue Zhang[1], Jun Zhang[1], Dongsheng Li[2], Hongguang Ren[1*], Junjie Xu[1*], Wei Chen[1*]

[1*]Laboratory of Advanced Biotechnology, Beijing Institute of Biotechnology, Beijing, China.

[2]College of Computer, National University of Defence Technology, Changsha, China.

*Corresponding author(s). E-mail(s): bioren@163.com; xujunjie@sina.com; cw0226@foxmail.com;

†These authors contributed equally to this work.

List of Supplementary information

Supplementary Figure 1. Taxonomic distribution of protective antigens across pathogen species.

Supplementary Figure 2. The metrics scores based on different feature dimensions for the sequence embeddings.

Supplementary Figure 3. Sequence alignment of MPXV G10R antigen and its ECTV homolog.

Supplementary Figure 4. G10R-specific monoclonal antibodies exhibit high-affinity binding and neutralizing activity against orthopoxviruses.

Supplementary Table 1. Performance of ESM-2 and the baselines on the constructed dataset.

Supplementary Table 2. Performance of different feature selection methods.

Supplementary Table 3. Performance of PLGDL and its variants trained on pathogen-specific subsets.

Supplementary Data 1. Protein information of the established antigen dataset.

Supplementary Data 2. AlphaFold3-predicted structural confidence metrics for antigen proteins.

Supplementary Data 3. Protein information of third-party dataset.

Supplementary Data 4. Protein information of Mpox virus.

Supplementary Data 5. Candidate antigens predicted for Mpox.

Supplementary Data 6. Homology of Mpox antigen G10R against human proteome.

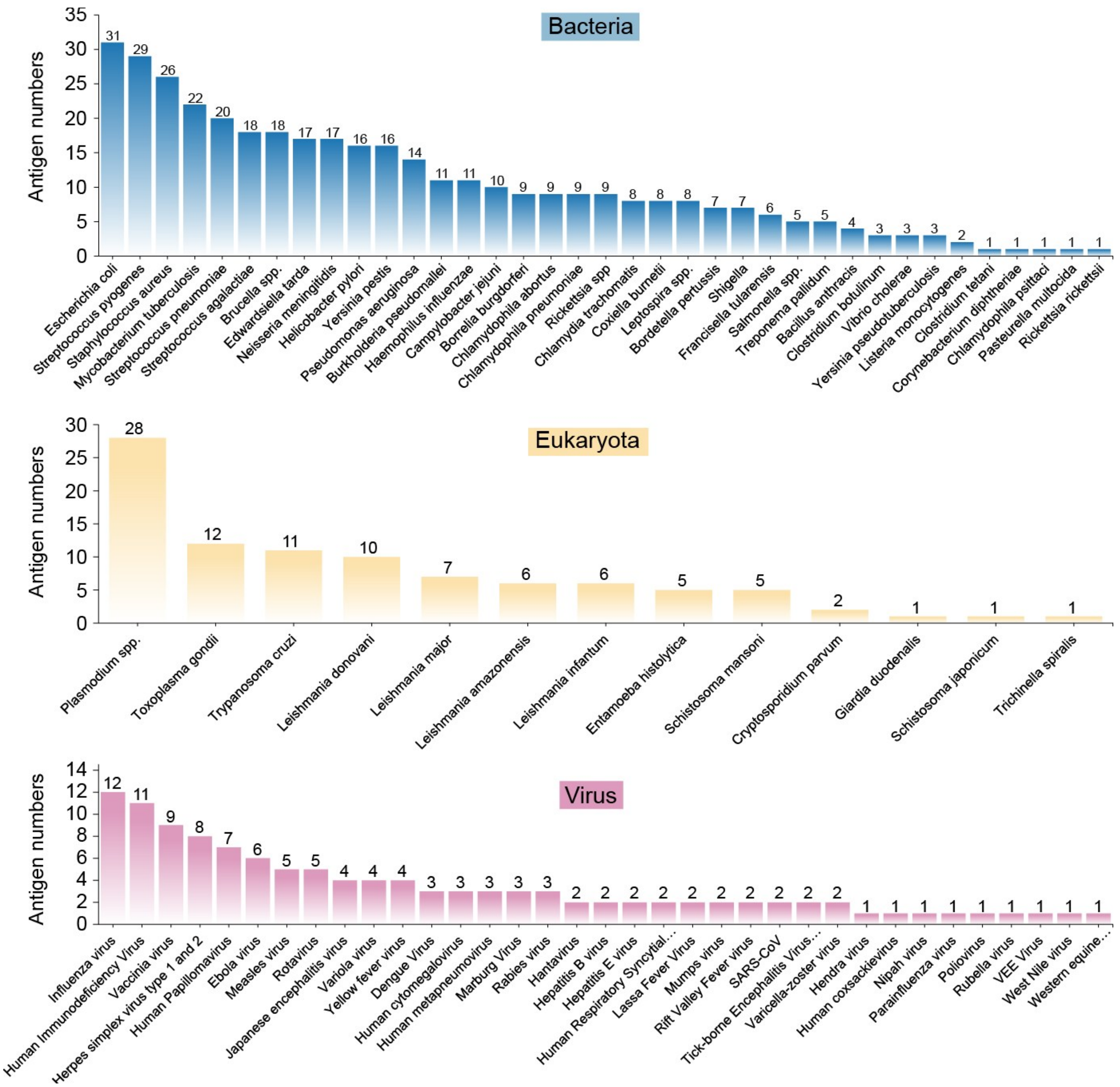

Supplementary Figure 1. Taxonomic distribution of protective antigens across pathogen species. Visual representation of protective antigen source diversity, with viral, bacterial, and eukaryotic pathogens ranked by their respective antigen representation in the database. Source data are provided as a Source Data file.

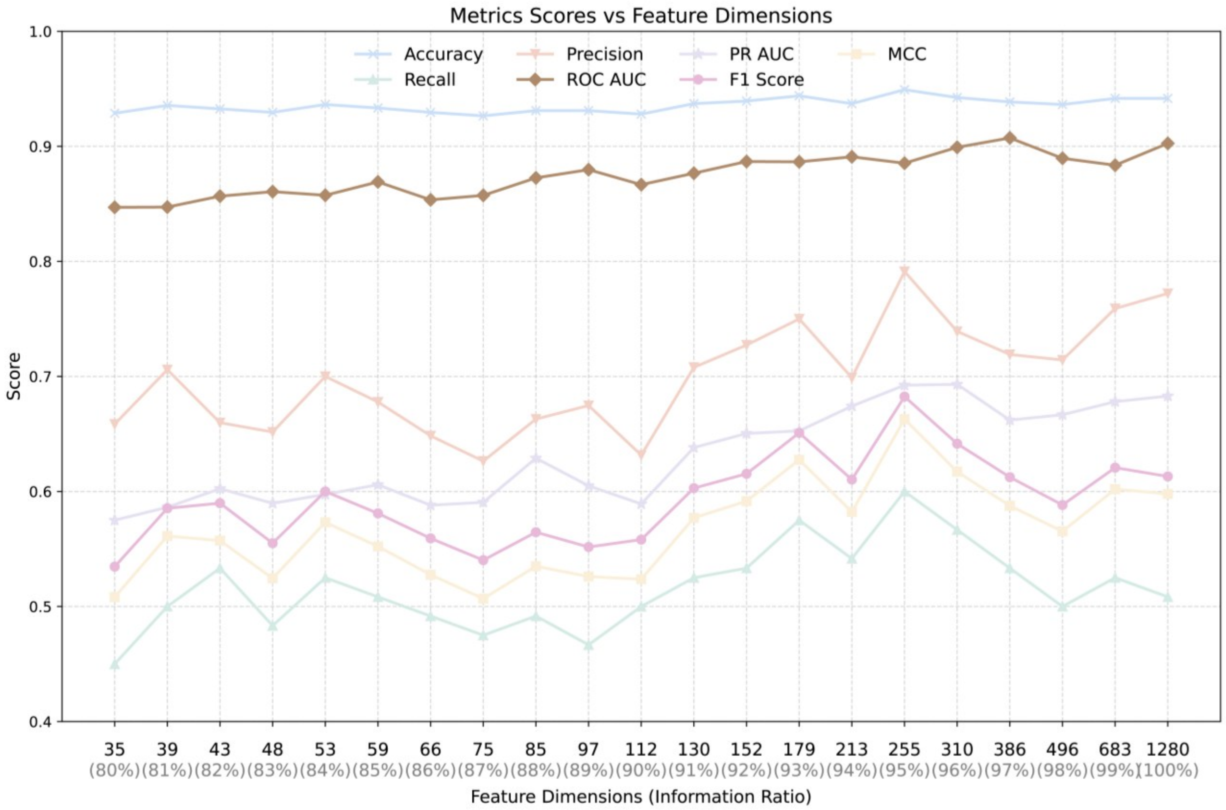

Supplementary Figure 2. The metrics scores based on different feature dimensions for the sequence embeddings. The figure illustrates the F1-score and MCC metrics at progressively reduced feature dimensions (from 1280D retaining 100% information to 59D retaining 85% information). The proposed 255-dimensional configuration achieves optimal performance, demonstrating an effective balance between information retention and feature redundancy elimination. Source data are provided as a Source Data file.

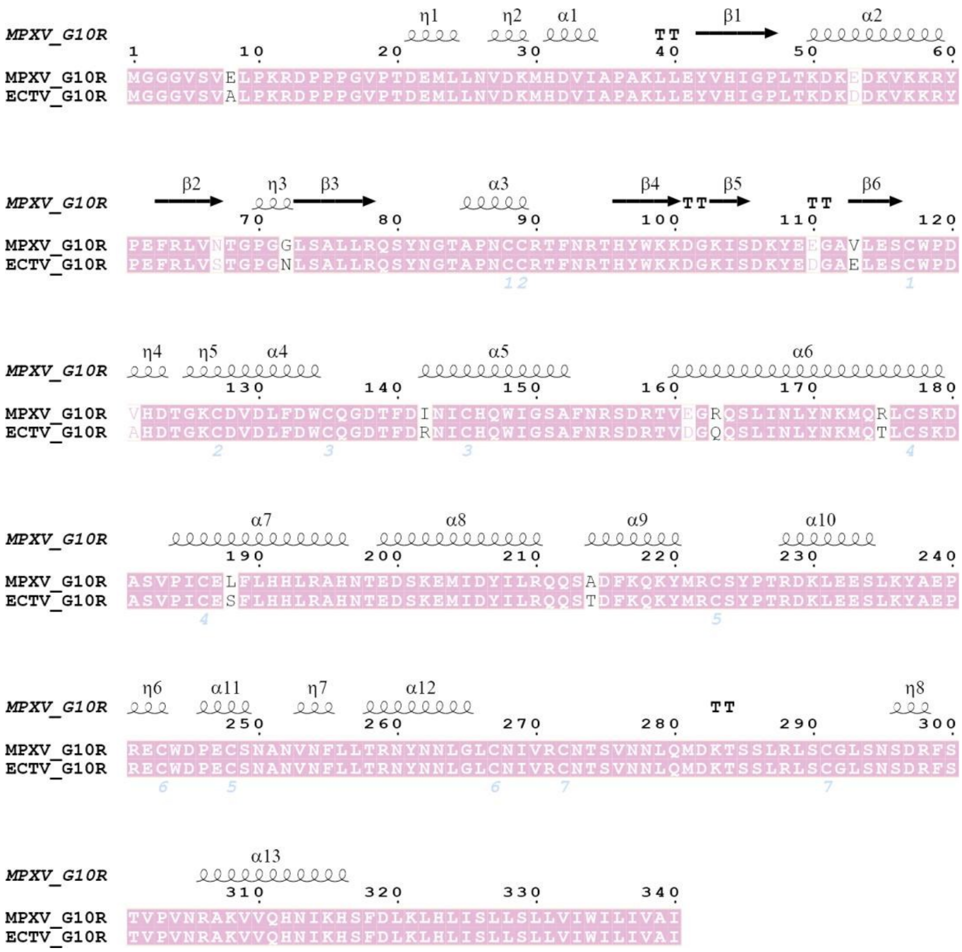

Supplementary Figure 3. Sequence alignment of MPXV G10R antigen and its ECTV homolog. The alignment reveals 96.47% amino acid identity (Clustal Omega), with secondary structural elements (α-helices, β-sheets) annotated.

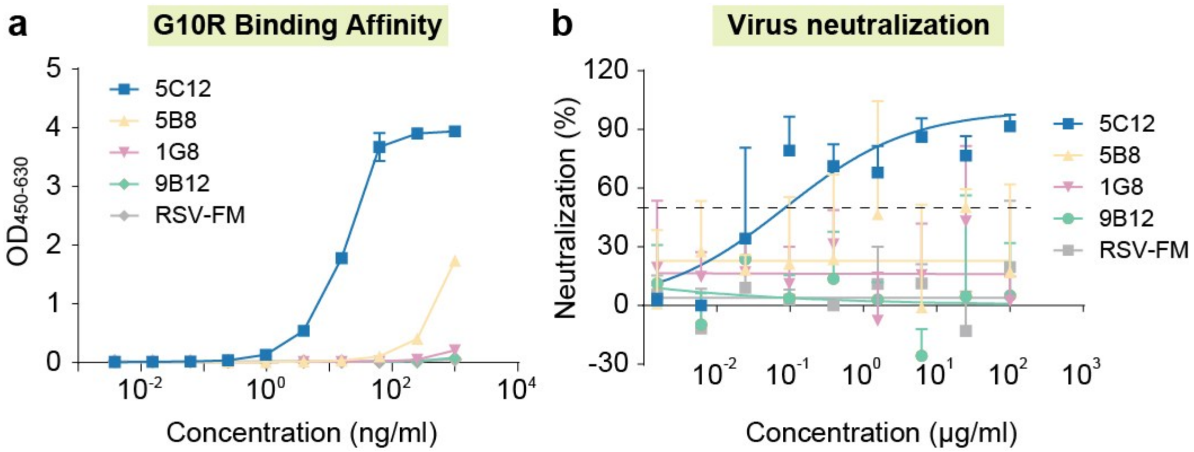

Supplementary Figure 4. G10R-specific monoclonal antibodies exhibit high-affinity binding and neutralizing activity against orthopoxviruses. a. Binding affinity of monoclonal antibodies to G10R antigen measured by ELISA. b, Neutralization of Vaccinia Virus by Monoclonal Antibodies. G10R-specific monoclonal antibodies (mAbs) were screened using hybridoma technology. Source data are provided as a Source Data file.

Supplementary Table 1. Performance of ESM-2 and the baselines on the constructed dataset

**Methods**	Accuracy	Precision	Recall	ROC-AUC	PR-AUC	F1 score	MCC
AMPLIFY	0.9167	0.5490	0.4667	0.8750	0.5327	0.5045	0.4611
Protrans	0.9415	0.7356	0.5333	**0.9065**	**0.7959**	0.6184	0.5957
ESM-2	**0.9492**	**0.7912**	**0.6000**	0.8853	0.6924	**0.6825**	**0.6629**

Note: The highest value for each metric is highlighted in bold.

Supplementary Table 2. Performance of different feature selection methods

**Methods**	Accuracy	Precision	Recall	ROC-AUC	PR-AUC	F1 score	MCC
SFS	0.9348	0.7500	0.4250	0.8669	0.6309	0.5426	0.5343
PCA	0.9394	0.7381	0.5167	0.8659	0.6289	0.6078	0.5869
RFE	0.9417	0.7590	0.5250	**0.9170**	**0.7035**	0.6207	0.6020
MI	0.9402	0.7253	0.5500	0.8795	0.6729	0.6256	0.6004
RF	0.9341	0.6854	0.5383	0.8896	0.6707	0.5837	0.5560
XGBoost	**0.9492**	**0.7912**	**0.6000**	0.8853	0.6924	**0.6825**	**0.6629**

Note: The highest value for each metric is highlighted in bold.

Supplementary Table 3. Performance of PLGDL and its variants trained on pathogen-specific subsets

Test Set	Training Set	Accuracy	Precision	Recall	F1 Score	ROC-AUC	PR-AUC	MCC
Bacteria	Bacteria only	**0.946**	0.688	0.571	0.624	**0.922**	0.660	0.598
	Virus+Bacteria+ Eukaryota	0.955	**0.804**	**0.577**	**0.672**	0.912	**0.708**	**0.658**
Eukaryota	Eukaryota only	0.942	0.688	0.579	0.629	**0.940**	0.685	0.600
	Virus+Bacteria+ Eukaryota	**0.954**	**0.769**	**0.588**	**0.667**	0.893	**0.718**	**0.649**
Virus	Virus only	0.867	**0.786**	0.458	0.579	0.764	0.583	0.532
	Virus+Bacteria+ Eukaryota	**0.872**	0.667	**0.640**	**0.653**	**0.872**	**0.727**	**0.575**

Note: The highest value for each metric is highlighted in bold.
