# Supplementary material for: Integrating protein language and geometric deep learning models for enhanced vaccine antigen prediction
Source: Nat Commun. 2025 Dec 21;17:1033. doi: 10.1038/s41467-025-67778-2 (PMC12847940; doi:10.1038/s41467-025-67778-2)
Supplement: Supplementary file 2 — Description of Additional Supplementary Files [file 41467_2025_67778_MOESM2_ESM.pdf]

Description of Additional Supplementary Files

Supplementary Data 1. Protein Information of the Established Antigen Dataset.

Supplementary Data 2. AlphaFold3-predicted structural confidence metrics for antigen proteins.

Supplementary Data 3. Protein Information of Third-Party Dataset.

Supplementary Data 4. Protein Information of Mpox Virus.

Supplementary Data 5. Candidate Antigens Predicted for Mpox.

Supplementary Data 6. Homology of Mpox antigen G10R against human proteome.
