# Supplementary material for: Integrating protein language and geometric deep learning models for enhanced vaccine antigen prediction
Source: Nat Commun. 2025 Dec 21;17:1033. doi: 10.1038/s41467-025-67778-2 (PMC12847940; doi:10.1038/s41467-025-67778-2)
Supplement: Supplementary file 9 — Reporting Summary [file 41467_2025_67778_MOESM9_ESM.pdf]

nature portfolio

Corresponding author(s): Wei Chen, Junjie Xu, Hongguang Ren

Last updated by author(s): Dec 1, 2025

Reporting Summary

Nature Portfolio wishes to improve the reproducibility of the work that we publish. This form provides structure for consistency and transparency in reporting. For further information on Nature Portfolio policies, see our Editorial Policies and the Editorial Policy Checklist.

Statistics

For all statistical analyses, confirm that the following items are present in the figure legend, table legend, main text, or Methods section.

n/a	Confirmed	
☐	☒	The exact sample size (*n*) for each experimental group/condition, given as a discrete number and unit of measurement
☐	☒	A statement on whether measurements were taken from distinct samples or whether the same sample was measured repeatedly
☐	☒	The statistical test(s) used AND whether they are one- or two-sided<br>*Only common tests should be described solely by name; describe more complex techniques in the Methods section.*
☐	☒	A description of all covariates tested
☐	☒	A description of any assumptions or corrections, such as tests of normality and adjustment for multiple comparisons
☐	☒	A full description of the statistical parameters including central tendency (e.g. means) or other basic estimates (e.g. regression coefficient) AND variation (e.g. standard deviation) or associated estimates of uncertainty (e.g. confidence intervals)
☐	☒	For null hypothesis testing, the test statistic (e.g. *F*, *t*, *r*) with confidence intervals, effect sizes, degrees of freedom and *P* value noted<br>*Give P values as exact values whenever suitable.*
☒	☐	For Bayesian analysis, information on the choice of priors and Markov chain Monte Carlo settings
☐	☒	For hierarchical and complex designs, identification of the appropriate level for tests and full reporting of outcomes
☒	☐	Estimates of effect sizes (e.g. Cohen's *d*, Pearson's *r*), indicating how they were calculated

Our web collection on statistics for biologists contains articles on many of the points above.

Software and code

Policy information about availability of computer code

Data collection	Protegen Database: Curated experimentally validated protective antigens. UniProt Database: Extracted pathogen protein sequences for negative dataset construction. AlphaFold Protein Structure Database: Provided high-quality predicted structures for graph-based modeling. PubMed: Identified newly reported vaccine antigens through literature mining.
Data analysis	We built and used PLGDL for data analysis. The code is available at: https://github.com/yunxiangz/PLGDL.<br>CD-HIT (v4.8.1): Redundancy reduction (50% sequence identity cutoff) for positive/negative datasets.<br>BLASTP (v2.14.0): Excluded negative dataset sequences with >20% homology to positive antigens.<br>AlphaFold3 (v3.0.1): Predicted 3D structures of antigens and non-antigens.<br>ESM-2 (esm2_t36_3B_UR50D): Generated 1,280-dimensional sequence embeddings (pre-trained on UniRef50) .<br>ProTrans: Produced 1,024-dimensional embeddings for sequence-structure relationships.<br>AMPLIFY: Integrated graph neural networks (GNNs) for hybrid sequence-structure embeddings.<br>GraphPad Prism 9.0.0: ANOVA, t-tests, survival curve analysis (log-rank test).<br>Microsoft Excel: Data organization and preliminary analysis.

For manuscripts utilizing custom algorithms or software that are central to the research but not yet described in published literature, software must be made available to editors and reviewers. We strongly encourage code deposition in a community repository (e.g. GitHub). See the Nature Portfolio guidelines for submitting code & software for further information.

Data

Policy information about availability of data

All manuscripts must include a data availability statement. This statement should provide the following information, where applicable:

- Accession codes, unique identifiers, or web links for publicly available datasets
- A description of any restrictions on data availability
- For clinical datasets or third party data, please ensure that the statement adheres to our policy

All data supporting the findings of this study, including raw datasets, processed results, and source data, are available within the main text, Supplementary Information, or Supplementary Data files.

Research involving human participants, their data, or biological material

Policy information about studies with human participants or human data. See also policy information about sex, gender (identity/presentation), and sexual orientation and race, ethnicity and racism.

Reporting on sex and gender	not applicable
Reporting on race, ethnicity, or other socially relevant groupings	not applicable
Population characteristics	not applicable
Recruitment	not applicable
Ethics oversight	not applicable

Note that full information on the approval of the study protocol must also be provided in the manuscript.

Field-specific reporting

Please select the one below that is the best fit for your research. If you are not sure, read the appropriate sections before making your selection.

☒ Life sciences ☐ Behavioural & social sciences ☐ Ecological, evolutionary & environmental sciences

For a reference copy of the document with all sections, see nature.com/documents/nr-reporting-summary-flat.pdf

Life sciences study design

All studies must disclose on these points even when the disclosure is negative.

Sample size	The animal study sample size of n = 6 mice per group was chosen based on previous, similar orthopoxvirus challenge studies and power calculations. No sample size calculation was performed for the in silico model development, as the largest available high-confidence dataset was used.
Data exclusions	No data were excluded from the analyses.
Replication	All experimental findings were successfully replicated. The key experiments, including the animal challenge studies, were repeated and yielded conclusions consistent with the original findings.
Randomization	Mice were allocated to experimental groups (n=6 per group) randomly upon arrival. Cages were assigned to treatment groups using simple randomization. All animals were female, age-matched, and sourced from the same vendor batch, minimizing covariates.
Blinding	Investigators were blind to group allocation during data collection and analysis.

Reporting for specific materials, systems and methods

We require information from authors about some types of materials, experimental systems and methods used in many studies. Here, indicate whether each material, system or method listed is relevant to your study. If you are not sure if a list item applies to your research, read the appropriate section before selecting a response.

Materials & experimental systems

n/a	Involved in the study	
☐	☒	Antibodies
☐	☒	Eukaryotic cell lines
☒	☐	Palaeontology and archaeology
☐	☒	Animals and other organisms
☒	☐	Clinical data
☒	☐	Dual use research of concern
☒	☐	Plants

Methods

n/a	Involved in the study	
☒	☐	ChIP-seq
☒	☐	Flow cytometry
☒	☐	MRI-based neuroimaging

Antibodies

Antibodies used	HRP-conjugated goat anti-mouse IgG (Abcam, ab97265, UK)
Validation	The secondary antibody (HRP-conjugated goat anti-mouse IgG, Abcam, Cat 97265) was validated by the manufacturer (Abcam) for use in ELISA. Specificity was confirmed by the vendor via western blot and ELISA against mouse IgG, with minimal cross-reactivity to other species.

Eukaryotic cell lines

Policy information about cell lines and Sex and Gender in Research

Cell line source(s)	BS-C-1 cells (ATCC, Cat CCL-26) were cultured with 10% fetal bovine serum (FBS, Thermo Scientific, USA), 1% penicillin, and 1% streptomycin in modified Eagle's medium (Thermo Fisher Scientific).
Authentication	Cell line identity was confirmed prior to experimental use.
Mycoplasma contamination	Cells were confirmed to be mycoplasma-free via PCR testing. Cell line identity was authenticated by the vendor (ATCC) via STR profiling prior to shipment. Cells were used within 10 passages of thawing.
Commonly misidentified lines (See ICLAC register)	BS-C-1 is not listed in the ICLAC Register of Misidentified Cell Lines.

Animals and other research organisms

Policy information about studies involving animals; ARRIVE guidelines recommended for reporting animal research, and Sex and Gender in Research

Laboratory animals	Specific pathogen-free (SPF) female BALB/c mice (6–8 weeks) were purchased from Charles River and were used for intramuscular immunization.
Wild animals	Did not involve wild animals.
Reporting on sex	Sex Consideration: Only female mice were used to minimize hormonal variability and align with historical data from analogous vaccine studies.<br>Sex-Based Analysis: No sex-based analyses were performed, as the experimental design intentionally excluded male mice to control for sex-related confounders.<br>Data Disaggregation: All data are derived from female mice; sex-specific data were not collected.
Field-collected samples	No field-collected samples were used in this study.
Ethics oversight	The experiments involving animals were approved and carried out according to the Institutional Animal Care and Use Committee guidelines of the Laboratory Animal Center (IACUC-DWZX-2024-P008).

Note that full information on the approval of the study protocol must also be provided in the manuscript.

Plants

Seed stocks	N/A.
Novel plant genotypes	N/A.
Authentication	N/A.
